# Interventions for Adjunctive Care in Patients With Inflammatory Bowel Disease and Permanent Ileostomy: A Systematic Review

**DOI:** 10.1093/crocol/otae056

**Published:** 2024-10-12

**Authors:** Sudheer Kumar Vuyyuru, Virginia Solitano, Yuhong Yuan, Neeraj Narula, Siddharth Singh, Christopher Ma, Florian Rieder, Vipul Jairath

**Affiliations:** Division of Gastroenterology, Western University, London, ON, Canada; Alimentiv Inc., London, ON, Canada; Division of Gastroenterology, Western University, London, ON, Canada; Division of Gastroenterology and Gastrointestinal Endoscopy, IRCCS Ospedale San Raffaele, Università Vita-Salute San Raffaele, Milan, Lombardy, Italy; Division of Gastroenterology, Western University, London, ON, Canada; Lawson Health Research Institute, London, Ontario, Canada; Division of Gastroenterology, Department of Medicine, Farncombe Family Digestive Health Research Institute, McMaster University, Hamilton, Ontario, Canada; Division of Gastroenterology, Department of Medicine, University of California, San Diego, La Jolla, CA, United States; Division of Gastroenterology, Western University, London, ON, Canada; Department of Community Health Sciences, Cumming School of Medicine, University of Calgary, Calgary, AB, Canada; Division of Gastroenterology and Hepatology, Department of Medicine, University of Calgary, Calgary, AB, Canada; Department of Inflammation and Immunity, Lerner Research Institute, Cleveland Clinic Foundation, Cleveland, OH, United States; Department of Gastroenterology, Hepatology and Nutrition, Digestive Diseases Institute, Cleveland Clinic Foundation, Cleveland, OH, United States; Program for Global Translational Inflammatory Bowel Diseases, Cleveland Clinic Foundation, Cleveland, OH, United States; Division of Gastroenterology, Western University, London, ON, Canada; Alimentiv Inc., London, ON, Canada; Department of Epidemiology and Biostatistics, Western University, London, ON, Canada

**Keywords:** permanent ileostomy, ostomy, stoma, inflammatory bowel disease, Crohn’s disease

## Abstract

**Background:**

The evidence for the management of patients with Crohn’s disease (CD) and permanent ileostomy (PI) is limited. We aimed to summarize the interventional studies related to the provision of adjunctive ostomy care in this population.

**Methods:**

MEDLINE, Embase, and Cochrane CENTRAL were searched from inception to January 5, 2024. Eligible studies were non-randomized or randomized controlled trials (RCTs), or comparative cohort studies predominantly recruiting participants with CD and/or ulcerative colitis (UC) with PI assessing interventions for the management of high stoma output, disease recurrence, peristomal skin care, pouching systems, behavioral interventions, mental health support, and diet.

**Results:**

Out of 3217 records, 6 were eligible and all were RCTs (*n* = 95). Out of these, 5 adopted a crossover design, and 1 study was a double-blind parallel-group RCT. All except 1 were published more than 20 years ago (1976-2003). Two studies exclusively included patients with UC, one included CD, and the remaining included both UC and CD. Four studies assessed pharmacological interventions (loperamide, 5-aminosalysilate [5-ASA], azodisal sodium, and budesonide), one assessed oral supplement with different osmolarities, and one assessed dietary intervention (unrefined vs refined carbohydrate). A decrease in ileostomy output was the primary outcome of interest in 4 studies. None of the studies assessed interventions for peristomal skin care, quality of life, stoma pouching systems, behavioral interventions, mental health, or CD recurrence.

**Conclusions:**

This study highlights that the evidence base to inform care for patients with IBD and PI is almost non-existent. There is an urgent need for focused research in this area to inform evidence-based treatment decisions.

## Introduction

The formation of an intestinal stoma is a surgical procedure involving the creation of an artificial opening in the abdominal wall that allows fecal flow to its outside and plays a pivotal role in managing various gastrointestinal conditions.^1^ It is estimated that more than 1 million people are currently living with a stoma in North America.^2^ While ileostomies can be temporary, specific indications warrant opting for a permanent ileostomy (PI) when the restoration of normal bowel function is unfeasible^3^ and/or determined by patient preference. Inflammatory bowel disease (IBD), including Crohn’s disease (CD), and ulcerative colitis (UC), is one such indication. Thirty to fifty percent of CD patients will require at least 1 surgery in their lifetime due to the consequences of uncontrolled inflammation.^4^ This includes the development of complications such as strictures, fistulae, or abscesses, often necessitating a PI. Patients with intestinal stoma experience stoma-related complications, including but not limited to skin changes due to leakage, stomal stenosis, ischemia/necrosis, hemorrhage, infection/abscess, a parastomal hernia, retraction/prolapse, and high stoma output.^5^ The cumulative probability of stoma-related complications in patients with CD and PI formation is 30.4% at 5 years, and 40.9% at 10 years.^6^ Another major concern in patients of CD with PI is postoperative recurrence. Up to one-third of CD patients can experience disease recurrence in the small intestine proximal to the PI within 10 years, typically requiring medical therapy; while 16% experience significant recurrence necessitating additional surgery.^7^ A meta-analysis of 18 cohort studies (1438 adults) who underwent subtotal or total colectomy or proctocolectomy with PI for colonic CD reported the cumulative risk of clinical recurrence surgical recurrence was 40% and 18.5% at 10 years, respectively.^8^ Moreover, clinical and surgical recurrence occurred in 11.5% and 10.4%, respectively, of patients without ileal disease at baseline.^8^

The current management principles for ostomy care for patients of CD with PI are based on evidence generated from studies that predominantly involved patients with malignancy. Moreover, despite progress in controlling intestinal inflammation with new drugs, patients with CD and PI are generally excluded from all drug trials.^9^ In a recent systematic review of over 80 randomized controlled trials (RCTs) evaluating the efficacy of advanced therapies, none included patients with PI.^9^ Thus there is a need for high-quality evidence to improve the quality of life in this group of patients.

The multicenter Endpoint Development for Ostomy Clinical Trial (Endo-trial) consortium, supported by the Helmsley Charitable Trust, is dedicated to addressing the gaps in care provision including the development of structured clinical care pathways for patients with CD and PI. As part of the workstreams, we aimed to comprehensively summarize key aspects of adjunctive care in patients with IBD and an ostomy. Specifically, we focused on existing interventional studies on the management of high stoma output, strategies to navigate disease recurrence, optimal approaches to peristomal skin care, considerations surrounding stoma pouching systems and adhesives, efficacy of behavioral interventions, avenues for mental health support, and strategies for dietary management.

## Methods

We conducted a systematic literature review on existing interventional studies for the management of high stoma output, strategies to navigate disease recurrence, optimal approaches to peristomal skin care, considerations surrounding stoma pouching systems and adhesives, the efficacy of behavioral interventions, avenues for mental health support, and strategies for dietary management in patients with IBD (CD or UC) with a PI. We followed the Cochrane Collaboration guideline for systematic review of interventional studies^10^ and the PRISMA (Preferred Reporting Items for Systematic Reviews and Meta-analyses) 2020 guideline for reporting systematic reviews.^11^ A review protocol was completed before starting the literature search and systematic review.

### Eligibility

Published RCTs, non-randomized clinical trials, and comparative cohort studies (prospective or retrospective) that reported data for patients with IBD with PI were eligible. We excluded studies that did not include a comparative arm (eg, single-arm cohort or case series) but included crossover studies in which two interventions were assessed one after another with a washout period. We included studies with any management of high stoma output, strategies to navigate disease recurrence, optimal approaches to peristomal skin care, considerations surrounding stoma pouching systems and adhesives, the efficacy of behavioral interventions, avenues for mental health support, and strategies for dietary management. Full text of studies that mixed patients with IBD and other indications, and studies including different types of ostomies (eg, cystostomy, colostomy, gastrostomy, urostomy, etc.) were screened for further information. We further excluded studies that did not provide separate data for patients with IBD or separate data for PI. We excluded studies that did not include patients with PI, (eg, only patients with temporary ileostomy, continent ileostomy, defunctioning loop stoma, permanent end colostomy, stoma closured, etc). We excluded studies that focused on diversion proctocolitis (DP) in IBD patients following fecal diversion (colostomy, ileostomy), and studies of surgical management of ostomy complications.

### Literature Search

We searched the following databases from inception to January 05, 2024: MEDLINE (Ovid, from 1946-); EMBASE (Ovid, from 1974-); Cochrane Central Register of Controlled Trials (CENTRAL) (Ovid, from inception). The search was limited to English language studies. Conference abstracts were excluded. Text words as well as MeSH or Emtree terms related to ostomy, ileostomy, and stoma were searched, combined with MeSH or Emtree terms related to IBD, CD, and UC, and combined with validated study design filters for RCTs and cohort studies. The search strategy can be found in Supplementary Appendix. A recursive manual search of the bibliography of eligible review articles and meta-analyses was conducted to ensure that all eligible systematic reviews were identified.

### Outcomes

Outcomes were documented and analyzed independently for each intervention. Some outcomes were reported involving different interventions. We pre-specified our outcomes of interest a priori in the protocol and assessed various outcome measures across different domains related to the clinical management of patients with an ileostomy. These outcomes were organized according to specific interventions and included the following: (1) for interventions related to stoma output: fecal output; (2) peristomal skin care: assessment of skin changes, and irritation as defined in the studies; (3) dietary management: fecal output or outcomes related to nutritional assessment; (4) interventions to improve quality of life and mental health: assessed by either stoma specific or general quality of life assessment tools and validated instruments for assessment of mental health; (5) educational interventions: stoma self-care ability, intimacy, quality of life, length of hospital stay, patient satisfaction; (6) stoma pouching systems and adhesives; and (7) interventions for prophylaxis or management of postoperative recurrence: proportion of patients with recurrence of CD as defined by the investigators.

### Data Extraction

References identified by the search strategy were imported into Covidence, each reference was screened by 3 reviewers [SKV, VS, and YY] independently and in duplicate, screened the title and abstract of all records. Two reviewers [YY, SV] independently screened full text and extracted data in duplicate. Disagreements between the reviewers were resolved through discussion. If consensus could not be reached, a third person [VJ] acted as the arbiter. A study-specific extraction form was used to collect data. The following data were extracted from the included studies: author, year of publication, study design, category of intervention, study interventions, IBD sample size, outcome data, etc.

### Risk of Bias Assessment

Cochrane risk of bias tool ROB 2 was used to assess the risk of bias of eligible RCTs.^12^ According to the Cochrane guidance, a variant of the RoB 2 tool specific to crossover trials was considered when assessing crossover RCTs.^13^ We planned to use the ROBINS-I tool (Risk Of Bias in Non-randomized Studies—of Interventions) to assess the risk of bias in non-randomized clinical trials or comparative cohort studies.^14^ Since no non-randomized clinical trials were included, only ROB 2 tool was applied.

### Statistical Analysis

In the absence of a sufficient number of eligible studies, we did not perform any statistical analysis.

## Results

**Figure 1.**
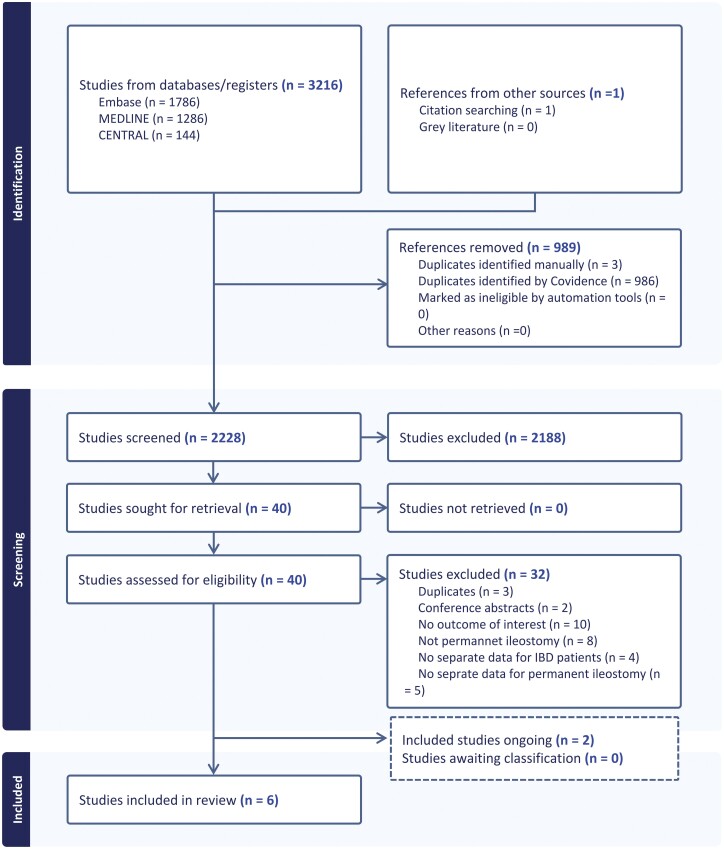
PRISMA flow diagram showing the selection of studies.

The literature search retrieved 3216 records (MEDLINE 1286, Embase 1786, and Cochrane CENTRAL 144), and 1 citation was identified from citation searching. After removing duplicates of 989 records, 2228 references were screened. Of these, 40 full-text papers were reviewed and 34 were considered ineligible (Supplementary Table 1). In total, 6 studies^15–20^ are included in this review (Figure 1).

All included studies were RCTs recruiting a total of 95 adult participants. Out of these, 5 studies adopted a crossover design and 1 study^19^ was a double-blind parallel-group RCT. Two studies exclusively included patients with UC^17,18^ and 1 study included patients with CD.^19^ Whereas, 3 studies included patients with both UC and CD.^15,16,20^ All except 1 study^20^ was published more than 20 years ago (1976-2003). Two studies assessed pharmacological interventions to reduce stoma output (loperamide and budesonide), 1 study assessed dietary intervention, 1 study assessed the effect of high and low osmolar oral supplements on stoma output, and the remaining 2 studies explored the effect of mesalamine on stoma output in patients with PI. None of the studies assessed interventions for peristomal skin care, quality of life, stoma pouching systems, or prophylaxis and/or management of postoperative recurrence in patients of CD and PI (characteristics of the included studies are presented in Table 1).

**Table 1.** Characteristics of interventional studies conducted in patients of inflammatory bowel disease and permanent ileostomy.

Authors, year, country	Study design	Number of participants	IBD subclass (*n*)	Intervention	Control	Washout period between two interventions	Outcomes	Conclusions
Tytgat 1976^15^ Netherlands	Double-blinded Placebo-controlled RCT Crossover design	20, ileostomy (total colonic resections with PI)	UC (12) CD (5) Polyposis coli (2) Adenocarcinoma (1)	Loperamide 8 mg/day (first 4 days) and were allowed to increase the dose to 12 mg/day (for the next 3 days)	Placebo	No washout period between loperamide and placebo	Ileostomy fecal output	Daily fecal outputs were significantly lower with loperamide compared to placebo
Berghouse 1984^16^ United Kingdom	Open-label Active comparator-controlled RCT Crossover design	10 patients with ileostomies	CD (5) UC (5)	Diet rich in refined carbohydrates	Diet with unrefined carbohydrates	One week	Microbial composition of ileostomy output	Approximately a 10-fold increase of total bacteria per gram of effluent when patients were on a diet with unrefined carbohydrates Significantly low wet and dry weight of ileostomy output with a diet rich in refined carbohydrates
Sandberg-Gertzen 1986^18^ Denmark	Double-blinded, placebo-controlled RCT Crossover design	8 patients who underwent colectomy with a PI	UC (8)	Azodisal sodium (1 g/day vs 2 g/day) or sulphasalazine (2 g/day) × 5 days	Placebo	Nine days	Ileostomy fluid output Prostaglandin levels in ileostomy output	Azodisal sodium but not sulfasalazine was associated with increased ileostomy output and was dose-related Azodisal sodium was not associated with increased prostaglandin levels in ileostomy output
Jarnerot 1987^17^ Sweden	Double-blinded, placebo-controlled RCT Crossover design	8 patients who underwent colectomy with a PI	UC (8)	Olsalazine sodium 0.75 g twice a day	Placebo	Seven weeks	Bile acids in ileostomy output	Bile acid excretion was not significantly different between Olsalazine and placebo
Ecker 2003^19^ Germany	Double-blinded Placebo-Controlled RCT, parallel-group design	40 patients with ileostomy and with a high ileostomy output (>1000 mL/24)	CD (40)	Oral budesonide 3 mg tid × 7 days	Placebo	NA	A >25% reduction in intestinal output during treatment was defined as the response	60% (12/20) of patients showed a response compared to none in the placebo group 30.2% decrease in median output was seen in the budesonide group compared to a 0.3% decrease in the placebo group
Rud 2019^20^	Double-blinded active comparator-controlled RCT Crossover design	8 patients with ileostomy and independent of parenteral nutrition	UC (5) CD (2) Cancer (1)	Iso-osmolar (279 mOsm/kg) oral supplement 200 mL × 4 times a day for 2 days	Hyperosmolar (681 mOsm/kg) oral supplement 200 mL × 4 times a day for 2 days	At least 14 days	Difference in ileostomy output	No statistically significant changes in ileostomy output. Iso-osmolar supplement induced a statistically significant increase in urine volume and natriuresis

Abbreviations: CD, Crohn’s disease; NA, not applicable; PI, permanent ileostomy; RCT, randomized controlled trial; UC, ulcerative colitis.

*Studies assessing stoma output as an outcome*

a) A study by Tytgat et al,^15^ was a double-blind, placebo-controlled, crossover study. Twenty patients with well-established ileostomy (UC: 12, CD: 5, polyposis coli: 2, and adenocarcinoma: 1) received either loperamide 2 mg for 7 days followed by placebo in sequence or in reverse order following an initial drug-free period of 3 days. Patients were instructed to take 2 capsules twice per day for the first 4 days followed by more if needed. Daily fecal output was significantly lower with loperamide compared to placebo. The median daily ileostomy output was 22% lower with loperamide (500 g [80-2010]) and 2% higher in patients with placebo (680 g [180-2020]) compared to the drug-free period (645 g [100-2620]).
b) A study by Sandberg-Gertzen et al,^18^ was a double-blind, randomized, placebo-controlled, crossover study recruiting 8 patients with UC with PI. The aim of the study was to explore the mechanism of diarrhea in patients intolerant to 5-ASAs. In the controlled study the volunteers were randomized in blocks of 4. During four different 5-day periods, one of the following was administered in gelatin capsules of identical appearance: azodisal sodium 2 g/day, azodisal sodium 1 g/day, sulphasalazine 2 g/day, or placebo. Ileostomy fluid output increased (*P* < .005) in a dose-related manner during intake of azodisal sodium (1 g/day vs 2 g/day) compared with placebo or sulphasalazine (2 g/day). The authors also measured the prostaglandin (PG) levels in ileostomy output but no significant changes in PG levels were observed.
c) A study by, Jarnerot et al,^17^ was a double-blinded, placebo-controlled, crossover, interventional study in which 8 patients with UC with PI were given olsalazine sodium (two 5-aminosalysilate [5-ASA] molecules linked by azo bond) 0.75 g twice daily or placebo for periods lasting 4 days each. Between the 2 medication periods, there was a washout period of 7 weeks. On day 3 of both test periods patients received selenium-labeled conjugated bile acid analogue. The aim of the investigation was to examine whether olsalazine affected the bile acid handling in the terminal ileum, which could result in diarrhea. Radioactivity of the excreted feces and ileostomy output were measured. Ileostomy output was statistically significantly increased during the olsalazine period compared with the placebo period (*P* = .014) however, there was no difference in bile acid excretion.
d) A study by Ecker et al,^19^ was a double-blinded placebo-controlled study with a parallel-group design. Forty CD patients with ileostomy who were in remission, after exclusion of inflammatory activity, but had high ostomy output were randomized to receive oral budesonide (9 mg/day) (*n* = 20) or placebo (*n* = 20) for 7 days. High ostomy output was defined as >1000 mL/day by self-report under non-controlled dietary conditions and no fluid restrictions. All patients underwent ileoscopy and biopsy prior to assessing for mucosal inflammation. A >25% reduction in intestinal output during treatment was defined as the response. The median absolute daily intestinal output in the budesonide decreased by 30.2% (1240-865 mL) from baseline compared to 0.3% (950-947.5 mL) in the placebo group. Notably, baseline stoma output was 1240 mL in the budesonide group compared to 950 mL in the placebo group. The authors attributed this difference to high baseline stoma output (>2000 mL) in 3 patients in the budesonide group. In the budesonide group, 60% (12/20) of patients showed a response compared to 0% (0/20) in patients randomized to placebo. Even though the authors included patients presumably in clinical remission, 5 patients in the budesonide group, and 3 patients in the placebo group had features of inflammation on ileoscopy.
e) A study by Rud et al,^20^ was a single-center, randomized, double-blinded, active comparator, crossover intervention study. The effect of iso-osmolar (279 mOsm/kg) and hyperosmolar (681 mOsm/kg) oral supplements on ileostomy output was investigated. 800 mL/day of oral supplements were administered during two separate 3-day intervention periods during which supplements were administered for 2 days along with habitual diet, with a washout period of at least 14 days separating the 2 treatment periods. No statistically significant changes in ileostomy output were detected following the intake of either oral supplement. Compared with the hyperosmolar supplement, the iso-osmolar supplement induced a statistically significant increase in urine volume and natriuresis.

*Studies assessing dietary intervention*

f) A study by Berghouse et al,^16^ was a randomized, open-label, crossover study, where 2 different diets were given to patients with ileostomy one after another in random order. A total of 10 patients (5 UC and 5 CD) with no evidence of clinical disease activity (for CD), although radiology was not performed, were given diet A (refined carbohydrates) followed by diet B (unrefined carbohydrates), each for 15 days or in reverse order with a gap of 1 week between the 2 diets. The amount of ileostomy effluent was greater while using the unrefined carbohydrate diet. The bacteriological flora per gram was also higher on the unrefined carbohydrate diet.

### Risk of Bias Assessment

Using the Cochrane RoB 2 tool, only one study was rated as low risk of bias^20^; 3 and 2 RCTs were rated as some concerns or high risk of bias, respectively (Supplementary Figure 1). Among the 5 crossover design RCTs, 1 open-label study,^15^ and 1 study without a washout period^16^ were considered at high risk of bias. For the other 3 studies, there were concerns due to the randomization process^17–19^ and potential carry-over effect^18^ (<14 days).

## Discussion

The creation of a PI is a life-changing event in patients with CD and more than one-third of patients will go on to develop stoma-related complications.^6,21,22^ There is a pressing need for high-quality evidence tailored to the needs of patients with CD with PI, with a goal to prevent and manage stoma-related complications effectively. We conducted a systematic search for interventional studies that focused on the management of stoma complications in patients with IBD who have undergone PI formation. We identified only 6 interventional studies and all except one study were conducted more than 2 decades ago. Five studies specifically assessed stoma output as an outcome measure. Two studies assessed pharmacological interventions to reduce stoma output and only 1 study assessed dietary intervention. This review highlights a major absence of evidence in the literature and underscores the need for high-quality research to inform clinical management.

A PI can affect various aspects of a patient’s life, including quality of life, sexual intimacy, self-esteem, employment, and social interactions, as well as impose a substantial financial burden.^23,24^ As a result, about two-thirds of patients avoid physical and social activities^25^; furthermore, patients with an intestinal stoma are at high risk of psychological co-morbidity such as adjustment disorder, depression, and anxiety.^26–28^ Thus the development of a standardized approach to ostomy care is highly desired to improve care for patients with a PI. Over the years, specialist ostomy care nurses have been implemented in specialized centers and there have been significant improvements in the quality of ostomy care.^29,30^ As a result, several expert consensus guidelines have issued recommendations on ostomy care and management of complications. However, the quality of evidence supporting these recommendations is low. For instance, in the recent Wound, Ostomy, and Continence Nurses Society (WOCN) consensus guidelines, the majority of the recommendations were based on level C evidence (2 or more case series with more than 10 patients).^31^ There is a paucity of research that has been conducted in patients with intestinal stoma, with the majority focusing on stoma pouching systems and surgical techniques.^32^

The underlying disease that necessitated ostomy surgery is also likely to influence the type of complications experienced, thereby affecting the patient’s quality of life. Inflammation due to postoperative recurrence in CD could be a key factor contributing to some of the stoma-related complications in this population. For example, in a retrospective study, 82% of reconstructed stomas due to stoma complications were shown to have evidence of recurrent CD on histopathological examination. Similarly, in a study by Ecker et al, the authors reported that two-thirds of resected ileostomies for stoma-related complications in patients with CD revealed inflammation on histopathological examination.^33^ Factors influencing quality of life and patients’ priorities and expectations vary depending on the underlying cause. For example, patients with CD may feel significant improvement in quality of life following PI especially if remission has been achieved.^34,35^ In a cross-sectional analysis, authors reported that even though an ostomy did not affect overall quality of life, it was associated with reduced social satisfaction.^35^ This finding could be because of assessment by using traditional quality-of-life instruments which are not designed and validated for patients for CD with PI. Therefore, appropriate tools to measure quality of life specific to patients with CD and PI should be developed and validated.

We did not find any interventional study on pre- and postoperative educational interventions, stoma pouching systems, and interventions for prophylaxis and/or management of postoperative CD recurrence. The role of pre- and postoperative educational interventions in preventing ostomy-related complications cannot be understated. Consequently, several expert consensus guidelines have recommended the incorporation of these interventions into ostomy care pathways.^31,36–38^ Appropriate pre- and postoperative educational interventions have been shown to be associated with better acceptability of the PI and are universally recommended by major international ostomy guidelines.^31,39,40^ Given the uniqueness of complications that may occur, educational interventions specific for patients with CD and PI should be incorporated into the ostomy care pathways. One of the common symptoms experienced by patients with ostomies is high stoma output which may cause electrolyte disturbance and require frequent hospital admissions and complications related to dehydration. Design and conduct of high-quality studies assessing interventions for high stoma output are challenging because of several underlying possible causes of high stoma output which could introduce heterogeneity into study design. Moreover, the definition of high output stoma is in itself heterogenous with no standard universally accepted definition of increased stoma output. We found only 2 studies designed to assess the efficacy of pharmacological therapies to reduce stoma output and only 1 study defined high stoma output as >1000 mL/day. Similarly, evidence supporting optimal dietary interventions in this population is sparse. The role of diet and the gut microbiome in the pathogenesis of CD is recognized.^41,42^ However, further studies are needed to explore their potential impact on disease recurrence and whether dietary or microbial manipulation therapies could prevent recurrence in the ileum proximal to the ostomy.

Another commonly experienced stoma related complications by patients with an ostomy regardless of the underlying cause are dermatological complications.^43^ While patients with CD and PI often face similar dermatological complications to those seen in patients who had stoma due to other conditions, complications such as pyoderma gangrenosum may be specifically associated with CD and may require immunosuppressive therapy.^44^ The current evidence on dermatological complications is primarily based on observational studies, highlighting the need for further research. Additionally, patients with CD and PI may require frequent reassessment of their stoma pouching systems due to rapid changes in weight and body contour, which commonly occur as their nutritional status improves following surgery. Therefore, patients with CD and PI require ostomy care pathways tailored to the needs specific to this population.

A unique issue specific to patients with CD and PI is postoperative recurrence.^45^ In the past 2 decades several effective advanced therapies were approved for the management of CD. However, no clinical trials evaluating the efficacy and safety of these advanced therapies have included patients with CD and PI.^9^ Therefore, there is a need to design and conduct pharmacological trials to include CD patients with a PI since the pathophysiology and response to therapy may be different compared to patients without PI. However, several barriers precluding the recruitment of these patients in CD trials need to be addressed. First, patients with CD and PI are generally considered difficult to treat as PI is usually considered a last resort after failure of available medical therapies. Second, the commonly used eligibility and outcome criteria for luminal CD clinical trials such as the CDAI, HBI, and patient-reported outcomes (PRO2) based on stool frequency may not be relevant to this group of patients, such that PROs specific to patients with PI should be developed. Third, traditional endoscopic disease activity measuring instruments such as the CD endoscopic index of severity (CDEIS), and simple endoscopic score for CD (SES-CD) have not been validated in patients of CD and PI. The partially validated Rutgeerts score to predict postoperative clinical recurrence following ileocolonic resection and primary anastomosis^46^ has not been systematically evaluated in patients with PI. Fourth, the operating properties of biomarkers including fecal calprotectin and C-reactive protein as outcome measures and those of imaging modalities such as small bowel ultrasound and magnetic resonance imaging scan are unclear in CD with PI. Finally, challenges in achieving adequate recruitment in clinical trials should also be considered.

A key finding from this review is an almost non-existent evidence base for the management of common complications of patients with CD living with PI. Furthermore, despite the considerable impact of complications related to CD necessitating a PI, we found a noticeable lack of evidence specifically addressing this subgroup. This review highlights significant gaps in the literature, emphasizing the critical necessity for the development of standardized clinical care approaches tailored specifically to this patient population. By addressing the identified gaps in the literature and prioritizing research efforts to generate high-quality evidence, clinicians and healthcare providers can better support the unique needs of this patient population and improve their quality of life.

## Supplementary Material

otae056_suppl_Supplementary_Materials

## Data Availability

The data supporting the findings of this study can be made available from the corresponding author upon reasonable request.
