# Supplementary material for: Interventions for Adjunctive Care in Patients With Inflammatory Bowel Disease and Permanent Ileostomy: A Systematic Review
Source: Crohns Colitis 360. 2024 Oct 12;6(4):otae056. doi: 10.1093/crocol/otae056 (PMC11503213; doi:10.1093/crocol/otae056)
Supplement: otae056_suppl_Supplementary_Materials [file otae056_suppl_supplementary_materials.docx]

**Supplement Figure 1: Risk of bias assessment**

| **Study** | **D1** | **DS** | **D2** | **D3** | **D4** | **D5** | **Overall** |
| --- | --- | --- | --- | --- | --- | --- | --- |
| Tytgat 1976^1^ |  |  |  |  |  |  |  |
| Berghouse 1984^2^ |  |  |  |  |  |  |  |
| Sandberg-Gertzen 1986^3^ |  |  |  |  |  |  |  |
| Jarnerot 1987^4^ |  |  |  |  |  |  |  |
| Ecker 2003^5^ |  | NA |  |  |  |  |  |
| Rud 2019^6^ |  |  |  |  |  |  |  |


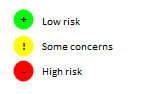


D1: Randomisation process; DS: Bias arising from period and carryover effects; D2: Deviations from the intended interventions; D3: Missing outcome data; D4: Measurement of the outcome; D5: Selection of the reported result

NA: Non-applicable. This study was not a crossover RCT, DS does not apply.

**Supplementary Table 1: Excluded studies with reasons**

| Sandberg-Gertzen 1986^3^ | Duplicate of already included study |
| --- | --- |
| Sandberg-Gertzen 1986^7^ | Duplicate of already included study |
| Ladefoged 1989^8^ | Not permanent ileostomy. Cohort study, no comparator |
| Levitt 1995^9^ | Not permanent ileostomy |
| Abrahamsson 1995^10^ | No outcome of interest |
| Abrahamsson 1995^11^ | Duplicate |
| Aman 1995^12^ | No outcome of interest |
| Ellegard 1997^13^ | No outcome of interest |
| Ellegard 2000^14^ | No outcome of interest |
| Cohen 2001^15^ | Not permanent ileostomy |
| Lundin 2004^16^ | No outcome of interest |
| Cragg 2005^17^ | No outcome of interest |
| ISRCTN45167712 | No available data |
| Ikeuchi 2006^18^ | No separate data for permanent ileostomy |
| Ellegard 2007^19^ | No outcome of interest |
| Funayama 2009^20^ | No separate data for permanent ileostomy |
| Banasiewicz 2013^21^ | Duplicate |
| Banasiewicz 2013^22^ | Not permanent ileostomy |
| Saito 2013^23^ | No outcome of interest |
| Kristensen 2017^24^ | No separate data for IBD patients |
| UMIN000015325 | Not permanent ileostomy |
| Uchino 2016^25^ | Not permanent ileostomy |
| Kwaan 2016^26^ | Conference abstract |
| Sier 2017^27^ | No separate data for IBD patients and PI |
| Iyer 2017^28^ | Conference abstract and no separate data for PI |
| Lightner 2018^29^ | No separate data for permanent ileostomy |
| Uchino 2018^30^ | Not permanent ileostomy |
| Grahn 2019^31^ | No separate data for IBD patients |
| Hubbard 2019^32^ | Protocol |
| ACTRN12620000166976 | No outcome of interest |
| Fiorindi 2021^33^ | No separate data for permanent ileostomy |
| Mesli 2021^34^ | No separate data for IBD patients |
| Lahes 2022^35^ | No separate data for permanent ileostomy |
| Tang 2023^36^ | Not permanent ileostomy |

**Supplementary Appendix:**

**Search strategy:**

**Database: Ovid MEDLINE(R) ALL <1946 to January 04, 2024>**

1. Ileostomy/
2. (Ileostomy or ileostomies).tw,kw,kf.
3. ((ostomy or ostomies) adj3 (entero* or instetinal* or ileo* or ileum or bowel or gut)).tw,kw,kf.
4. ((stoma or stomas) adj3 (entero* or instetinal* or ileo* or ileum or bowel or gut or abdominal or abdomen)).tw,kw,kf.
5. or/1-4
6. exp Inflammatory Bowel Diseases/
7. (ulcerative colitis or ulcerative colorectitis or proctocolitis or ulcerative enteritis or regional enteritis or Colorectitis or Ileocolitis).tw,kw,kf.
8. crohn*.tw,kw,kf.
9. (IBD or inflammatory bowel disease*).tw,kw,kf.
10. or/6-9
11. 5 and 10
12. exp randomized controlled trial/
13. controlled clinical trial.pt.
14. random*.ab.
15. placebo.ab.
16. drug therapy.fs.
17. trial.ab.
18. groups.ab.
19. exp cohort studies/
20. (cohort* or follow-up or followup or longitudinal* or prospective* or retrospective*).ti,ab.
21. epidemiologic methods/
22. or/12-21
23. exp animals/ not humans.sh.
24. 22 not 23
25. 11 and 24
26. limit 25 to english language

Note: Lines12-18, Cochrane Highly Sensitive Search Strategy for identifying randomized trials in MEDLINE: sensitivity-maximizing version (2023 revision); Ovid format (<https://training.cochrane.org/handbook/current/chapter-04>). Lines 19-21, adapted from BMJ Medline cohort study strategy ( <https://bestpractice.bmj.com/info/toolkit/learn-ebm/study-design-search-filters/> )

**Cochrane Central Register of Controlled Trials <December 2023>,**

1. Ileostomy/
2. (Ileostomy or ileostomies).tw,kw,kf.
3. ((ostomy or ostomies) adj3 (entero* or instetinal* or ileo* or ileum or bowel or gut)).tw,kw,kf.
4. ((stoma or stomas) adj3 (entero* or instetinal* or ileo* or ileum or bowel or gut or abdominal or abdomen)).tw,kw,kf.
5. or/1-4
6. exp Inflammatory Bowel Diseases/
7. (ulcerative colitis or ulcerative colorectitis or proctocolitis or ulcerative enteritis or regional enteritis or Colorectitis or Ileocolitis).tw,kw,kf.
8. crohn*.tw,kw,kf.
9. (IBD or inflammatory bowel disease*).tw,kw,kf.
10. or/6-9
11. 5 and 10

**Embase <1974 to 2024 January 04>,**

1. ileostomy/
2. (Ileostomy or ileostomies).tw,kw,kf.
3. ((ostomy or ostomies) adj3 (entero* or instetinal* or ileo* or ileum or bowel or gut)).tw,kw,kf.
4. ((stoma or stomas) adj3 (entero* or instetinal* or ileo* or ileum or bowel or gut or abdominal or abdomen)).tw,kw,kf.
5. or/1-4
6. exp inflammatory bowel disease/
7. (ulcerative colitis or ulcerative colorectitis or proctocolitis or ulcerative enteritis or regional enteritis or Colorectitis or Ileocolitis).tw,kw,kf.
8. crohn*.tw,kw,kf.
9. (IBD or inflammatory bowel disease*).tw,kw,kf.
10. or/6-9
11. 5 and 10
12. exp randomized controlled trial/
13. Controlled clinical trial/
14. random$.ti,ab.
15. randomization/
16. intermethod comparison/
17. placebo.ti,ab.
18. (compare or compared or comparison).ti.
19. ((evaluated or evaluate or evaluating or assessed or assess) and (compare or compared or comparing or comparison)).ab.
20. (open adj label).ti,ab.
21. ((double or single or doubly or singly) adj (blind or blinded or blindly)).ti,ab.
22. double blind procedure/
23. parallel group$1.ti,ab.
24. (crossover or cross over).ti,ab.
25. ((assign$ or match or matched or allocation) adj5 (alternate or group$1 or intervention$1 or patient$1 or subject$1 or participant$1)).ti,ab.
26. (assigned or allocated).ti,ab.
27. (controlled adj7 (study or design or trial)).ti,ab.
28. (volunteer or volunteers).ti,ab.
29. human experiment/
30. trial.ti.
31. or/12-30
32. (random$ adj sampl$ adj7 ("cross section$" or questionnaire$1 or survey$ or database$1)).ti,ab. not (comparative study/ or controlled study/ or randomi?ed controlled.ti,ab. or randomly assigned.ti,ab.)
33. Cross-sectional study/ not (exp randomized controlled trial/ or controlled clinical study/ or controlled study/ or randomi?ed controlled.ti,ab. or control group$1.ti,ab.)
34. (((case adj control$) and random$) not randomi?ed controlled).ti,ab.
35. Systematic review.ti,ab. not (trial or study).ti.
36. (nonrandom$ not random$).ti,ab.
37. "random field$".ti,ab.
38. (random cluster adj3 sampl$).ti,ab.
39. (review.ab. and review.pt.) not trial.ti.
40. "we searched".ab. and (review.ti. or review.pt.)
41. "update review".ab.
42. (databases adj4 searched).ab.
43. (rat or rats or mouse or mice or swine or porcine or murine or sheep or lambs or pigs or piglets or rabbit or rabbits or cat or cats or dog or dogs or cattle or bovine or monkey or monkeys or trout or marmoset$1).ti. and animal experiment/
44. Animal experiment/ not (human experiment/ or human/)
45. or/32-44
46. 31 not 45
47. cohort analysis/
48. longitudinal study/
49. prospective study/ or retrospective study/
50. follow up/
51. (cohort* or follow-up or followup or longitudinal* or prospective* or retrospective*).ti,ab.
52. or/47-51
53. 52 not (43 or 44)
54. 46 or 53
55. 11 and 54
56. (conference abstract or Conference Review).pt.
57. 55 not 56
58. limit 57 to english language

Note: Lines 12-46. Cochrane Highly Sensitive Search Strategy for identifying randomized trials in Embase (2023 revision); Ovid format. (<https://training.cochrane.org/handbook/current/chapter-04>) Lines 47-52. Adapted from BMJ Embase cohort study strategy ( <https://bestpractice.bmj.com/info/toolkit/learn-ebm/study-design-search-filters/> )

**REFERENCES**

1. Tytgat GN, Huibregtse K, Meuwissen SG. Loperamide in chronic diarrhea and after ileostomy: A placebo-controlled double-blind cross-over study. *Arch Chir Neerl* 1976;**28**:13-20.

2. Berghouse L, Hori S, Hill M*, et al.* Comparison between the bacterial and oligosaccharide content of ileostomy effluent in subjects taking diets rich in refined or unrefined carbohydrate. *Gut* 1984;**25**:1071-7.

3. Sandberg-Gertzén H, Järnerot G, Bukhave K, Lauritsen K, Rask-Madsen J. Effect of azodisal sodium and sulphasalazine on ileostomy output of fluid and pge2 and pgf2 alpha in subjects with a permanent ileostomy. *Gut* 1986;**27**:1306-11.

4. Järnerot G, Sandberg-Gertzén H, Tobiasson P, Vikterlöf KJ. Olsalazine does not increase the bile acid losses in subjects with a permanent ileostomy. *Scand J Gastroenterol* 1988;**23**:489-92.

5. Ecker KW, Stallmach A, Seitz G*, et al.* Oral budesonide significantly improves water absorption in patients with ileostomy for crohn disease. *Scand J Gastroenterol* 2003;**38**:288-93.

6. Rud C, Pedersen AKN, Wilkens TL*, et al.* An iso-osmolar oral supplement increases natriuresis and does not increase stomal output in patients with an ileostomy: A randomised, double-blinded, active comparator, crossover intervention study. *Clin Nutr* 2019;**38**:2079-86.

7. Sandberg-Gertzen H, Jarnerot G, Bukhave K, Lauritsen K, Rask-Madsen J. Effect of azodisal sodium and sulphasalazine on ileostomy output of fluid and pge2 and pgf2 alpha in subjects with a permanent ileostomy. *Gut* 1986;**27**:1306-11.

8. Ladefoged K, Christensen KC, Hegnhoj J, Jarnum S. Effect of a long acting somatostatin analogue sms 201-995 on jejunostomy effluents in patients with severe short bowel syndrome. *Gut* 1989;**30**:943-9.

9. Levitt MD, Jenner DC, Maher MJ. High dose loperamide suppositories: A novel approach for improving clinical function after restorative proctocolectomy. *The Australian and New Zealand journal of surgery* 1995;**65**:881-3.

10. Abrahamsson M, Aman P, Hallmans G, Zhang JX, Lundin E. Excretion of amino acid residues from diets based on low-fibre wheat or high-fibre rye bread in human subjects with ileostomies. *European journal of clinical nutrition* 1995;**49**:589-95.

11. Abrahamsson M, Aman P, Hallmans G, Zhang JX, Tidehag P. Excretion of amino acid residues from diets based on wheat flour or oat bran in human subjects with ileostomies. *European journal of clinical nutrition* 1995;**49**:596-604.

12. P. A, D. P, J.-X. Z, P. T, G. H. Starch and dietary fiber components are excreted and degraded to variable extents in ileostomy subjects consuming mixed diets with wheat- or oat-bran bread. *Journal of Nutrition* 1995;**125**:2341-7.

13. Ellegard L, Andersson H, Bosaeus I. Inulin and oligofructose do not influence the absorption of cholesterol, or the excretion of cholesterol, ca, mg, zn, fe, or bile acids but increases energy excretion in ileostomy subjects. *European journal of clinical nutrition* 1997;**51**:05-Jan.

14. L. E, I. B, H. A. Will recommended changes in fat and fibre intake affect cholesterol absorption and sterol excretion? An ileostomy study. *European Journal of Clinical Nutrition* 2000;**54**:306-13.

15. Cohen LD, Levitt MD. A comparison of the effect of loperamide in oral or suppository form vs placebo in patients with ileo-anal pouches. *Colorectal disease : the official journal of the Association of Coloproctology of Great Britain and Ireland* 2001;**3**:Sep-95.

16. Lundin EA, Zhang JX, Lairon D*, et al.* Effects of meal frequency and high-fibre rye-bread diet on glucose and lipid metabolism and ileal excretion of energy and sterols in ileostomy subjects. *European journal of clinical nutrition* 2004;**58**:1410-9.

17. Cragg RA, Phillips SR, Piper JM*, et al.* Homeostatic regulation of zinc transporters in the human small intestine by dietary zinc supplementation. *Gut* 2005;**54**:469-78.

18. Ikeuchi H, Yamamura T, Kusunoki M*, et al.* Leukocyte removal therapy for ulcerative colitis does not affect postoperative complications. *Journal of gastroenterology* 2006;**41**:848-54.

19. L. E, H. A. Oat bran rapidly increases bile acid excretion and bile acid synthesis: An ileostomy study. *European Journal of Clinical Nutrition* 2007;**61**:938-45.

20. Funayama Y, Kumagai E, Takahashi K-I, Fukushima K, Sasaki I. Early diagnosis and early corticosteroid administration improves healing of peristomal pyoderma gangrenosum in inflammatory bowel disease. *Diseases of the colon and rectum* 2009;**52**:311-4.

21. Banasiewicz T, Horbacka K, Karon J*, et al.* Preliminary study with sprayshield tm adhesion barrier system in the prevention of abdominal adhesions. *Wideochirurgia i inne techniki maloinwazyjne = Videosurgery and other miniinvasive techniques* 2013;**8**:301-9.

22. T B, K H, J K*, et al.* Preliminary study with sprayshield&trade; adhesion barrier system in the prevention of abdominal adhesions. 2013;**8**:301.

23. Toru Saito KS, Keiji Koda, Kenji Oda, Takashi Shida, Masaru Miyazaki. Effects of intravenous cyclosporin on the surgical treatment for acute severe ulcerative colitis. *Journal of Gastroenterology and Hepatology Research* 2013; **2**:814-7.

24. Kristensen K, Qvist N. The acute effect of loperamide on ileostomy output: A randomized, double-blinded, placebo-controlled, crossover study. *Basic Clin Pharmacol Toxicol* 2017;**121**:493-8.

25. Uchino M, Hirose K, Bando T*, et al.* Randomized controlled trial of prophylactic negative-pressure wound therapy at ostomy closure for the prevention of delayed wound healing and surgical site infection in patients with ulcerative colitis. *Digestive surgery* 2016;**33**:449-54.

26. MR K, SW G, AC L*, et al.* Education program for prevention of ileostomy complications (eppic): A randomized trial. 2016;**223**:e7.

27. M.F. S, R.J. O, M.G.W. D*, et al.* Home visits as part of a new care pathway (iaid) to improve quality of care and quality of life in ostomy patients: A cluster-randomized stepped-wedge trial. *Colorectal Disease* 2017;**19**:739-49.

28. K I, PB J, SM G*, et al.* Patients with short bowel syndrome stratified by diagnosis: Post hoc analysis of teduglutide on fluid composite effect. 2017;**112**:S661.

29. Lightner AL, Tse CS, Potter DDJ, Moir C. Postoperative outcomes in vedolizumab-treated pediatric patients undergoing abdominal operations for inflammatory bowel disease. *Journal of pediatric surgery* 2018;**53**:1706-9.

30. Uchino M, Ikeuchi H, Bando T*, et al.* Ostomy creation with fewer sutures using tissue adhesives (cyanoacrylates) in inflammatory bowel disease: A pilot study. *Annals of the Royal College of Surgeons of England* 2018;**100**:190-3.

31. Grahn SW, Lowry AC, Osborne MC*, et al.* System-wide improvement for transitions after ileostomy surgery: Can intensive monitoring of protocol compliance decrease readmissions? A randomized trial. *Diseases of the colon and rectum* 2019;**62**:363-70.

32. Hubbard G, Beeken RJ, Taylor C*, et al.* A physical activity intervention to improve the quality of life of patients with a stoma: A feasibility study protocol. *Pilot and Feasibility Studies* 2019;**5**:78.

33. Fiorindi C, Cuffaro F, Piemonte G*, et al.* Effect of long-lasting nutritional prehabilitation on postoperative outcome in elective surgery for ibd. *Clinical nutrition (Edinburgh, Scotland)* 2021;**40**:928-35.

34. Y. M, L. H, J.-B. D*, et al.* Is lanreotide really useful in high output stoma? Comparison between lanreotide to conventional antidiarrheal treatment alone. *Journal of Investigative Surgery* 2021;**34**:1312-6.

35. S. L, C. F, A.E. S*, et al.* Effect of immunosuppressive medication on postoperative complications following abdominal surgery in crohn's disease patients. *International Journal of Colorectal Disease* 2022;**37**:2535-42.

36. Tang Z, Sun S, Ji M*, et al.* Long-term outcomes after enterostomy for very early-onset inflammatory bowel disease with interleukin-10 signaling deficiency. *BMC gastroenterology* 2023;**23**:404.
